# Alternative Anesthesia of Neonatal Mice for Global rAAV Delivery in the Brain With Non-detectable Behavioral Interference in Adults

**DOI:** 10.3389/fnbeh.2020.00115

**Published:** 2020-07-14

**Authors:** Wannan Tang, Uwe Zillmann, Rolf Sprengel

**Affiliations:** ^1^Department of Molecular Neurobiology, Max Planck Institute for Medical Research, Heidelberg, Germany; ^2^GliaLab and Letten Centre, Department of Molecular Medicine, Division of Physiology, Institute of Basic Medical Sciences, University of Oslo, Oslo, Norway; ^3^Department of Clinical and Molecular Medicine, Norwegian University of Science and Technology, Trondheim, Norway; ^4^Research Group of the Max Planck Institute for Medical Research, Institute for Anatomy and Cell Biology, Heidelberg University, Heidelberg, Germany

**Keywords:** anesthesia, medetomidine, midazolam, fentanyl, neonatal gene delivery, rAAV gene transduction, behavioral tests

## Abstract

Viral-transduced gene expression is the current standard for cell-type-specific labeling and cell tacking in experimental neuroscience. To achieve widespread gene expression, a viral delivery method to neonatal rodents was introduced more than two decades ago. Most of those neonatal viral vector injection-based gene transduction methods in mice used deep hypothermia for anesthesia, which was reported to be associated with behavioral impairments. To explore other options for neonatal viral applications, we applied a combination of Medetomidine, Midazolam, and Fentanyl (MMF), each of which can be antagonized by a specific antagonist. Later in their adulthood, we found that adult mice, that received the MMF-induced anesthesia, combined with virus-injected into the brain at postnatal day 2, showed similar performance in all behavioral tasks tested, including tasks for motor coordination, anxiety-related tasks, and spatial memory when compared to adult naïve littermates. This demonstrates that MMF anesthesia could be safely applied to mice for neonatal viral transduction at P2.

## Introduction

Recombinant adeno-associated virus (rAAV) injection in specific brain areas is now widely used to achieve specific gene transduction in various brain regions and neuronal and glia populations. Classical virus-mediated gene transfer approaches into pre-selected brain regions are mostly limited to adult animals *via* stereotactic injections (Cetin et al., 2007). For a global expression of transduced genes in the mouse brain, a neonatal rAAV delivery method was introduced and used successfully (Passini and Wolfe, 2001; Pilpel et al., 2009; Chakrabarty et al., 2013; Kim et al., 2013, 2014; McLean et al., 2014; Ayers et al., 2015; He et al., 2018). As alternatives to this global, neonatal rAAV gene transduction, *in utero* electroporation (Saito, 2006; Huang and Carcagno, 2018), or systemic injection of rAAV into the tail vein of adult or adolescent mice (Foust et al., 2009; Stoica et al., 2013; Körbelin et al., 2016; Thomsen et al., 2017) achieved similar expression of transfected or transduced genes in the mouse brain. However, high expression of the transfected gene is of relatively short duration after *in utero* electroporation and is more restricted to the area that received the DNA injection. For successful tail vain delivery, high titer rAAVs and large injection volumes are necessary to achieve a widespread rAAV infection in the brain, and the function of the transduced gene cannot be studied during the early development of the nervous system.

Therefore, we used the neonatal rAAV gene delivery method in a pilot study with cryoanesthesia, also referred to as hypothermia, to establish a mouse model for autism spectrum disorder (ASD; Berkel et al., 2012). In this anesthesia method, which was initially introduced for neonatal rats (Wiesner, 1934), the newborn animals are separated from their mothers and immobilized on ice for 3–5 min before the virus injection. However, two studies with human patients have shown that hypothermia is associated with wound infections, increased operative blood loss, and other complications (Kurz et al., 1995; Tanaka et al., 2001). In experimental animals, similar complications were found, such as apnea, hypoxia (Adolph, 1948), and slightly increased anxiety (Richter et al., 2014), which might have long-term impacts on the behavioral tests of cryoanesthetized neonates. Although cryoanesthesia is a frequently accepted method that provides immobility and mild analgesia, an increasing number of studies have evaluated cryoanesthesia as harmful and unacceptable for being applied in pets, working animals, and animals in experimental research.

We received the governmental permission to investigate the application and long-term effects of a “new” set of general anesthesia drugs in newborn mice. We decided to use a the combination of Medetomidine (M-Domitor), Midazolam (M-Dormicum) and Fentanyl (F-Fentanyl; in short MMF), since (I) each of the three drugs can be antagonized by a very specific antagonist, and (II) MMF anesthesia had been used for rabbits and other mammals (Henke et al., 2005). Medetomidine and its specific antagonist Atipamezole (A-Antiseden) react in the central and peripheral nervous system with β_2_-adrenoceptors, Midazolam and its antagonist Flumazenil (F-Anexate) bind to GABA_A_ receptors, while Fentanyl and its antagonist Naloxone (N-Narcanti) act on μ-opioid receptors. These drugs and their antagonists can be injected either intramuscularly or subcutaneously. The half-life of these compounds is between 0.5 and 3.0 h. Although this combination is one of the well-established anesthetic techniques, it is not yet commonly used and not everywhere accepted for small rodents. Moreover, it is not known how this pharmacological intervention that takes place shortly after birth affects later the behavior and cognitive performance of those mice.

In this study, we administrated the combination of MMF followed by their antagonists Atipamezole/Flumazenil/Naloxone (in short AFN) to the neonatal C57BL/6N and FVB/N mice and determined the earliest time point for successful pharmacological MMF-AFN anesthesia combined with rAAV injection. Later in adulthood of MMF-AFN/rAAV anesthetized mice, the locomotion, anxiety and cognitive performance were evaluated.

## Materials and Methods

### Ethics Statement

All experimental procedures were performed according to the animal welfare guidelines of the Max Planck Society and were in accordance with the German Animal Protection Law (TierSCHG). The Animal Ethics Committee of the MPImF/Heidelberg approved all experimental procedures and the regional veterinary authorities in Karlsruhe, Germany (License Number: 35-9185.82/A-38/10) accepted and supported the experimental protocol. The project was in accordance with the European 2006 Convention ETS 123 EG and Parliament/Council Directive 2010/63/EU. Legal access to drug was approved by the BfAM license to UZ.

### Animals and Administration of Anesthetics and Antagonists

Pregnant embryonic day 16 (E16) C57BL/6N and FBV/N mice were purchased from Charles River. Pups were born 5–6 days later at the animal facility of the Max Planck Institute for Medical Research. On the day of treatment, the postnatal day 0–2 (P0–P2) pups were first removed from the home cages and their dams. Pups were immediately anesthetized with a mixture of Medetomidine (M-Domitor), Midazolam (M-Dormicum) and Fentanyl (F-Fentanyl) [MMF mixture], which was prepared by 50 μl Medetomidine (1 mg/ml, Pfizer), 100 μl Midazolam (5 mg/ml, Pfizer) and 100 μl Fentanyl (0.05 mg/ml, Pfizer). Except controls, each pup was injected subcutaneously in the area of the left hip with 2.5 μl MMF/g body weight (dosage/kg of body weight: Medetomidine 0.5 mg/kg, Midazolam 5 mg/kg and Fentanyl 0.05 mg/kg). For the P2 pups, 5–10 min after MMF application, the deeply anesthetized animals were injected with serotype 1/2 rAAV-*SYN*-Venus. Up to 30 min after MMF treatment, the AFN antagonists [50 μl Atipamezol (5 mg/ml, Pfizer) + 500 μl Flumazenil (0.1 mg/ml, Pfizer) + 300 μl Naloxon (0.4 mg/m, Pfizer)] were injected subcutaneously into the right hip (8.5 μl/g body weight). The pups recovered about 3 min after the application of the AFN mixture.

### Stereotactic Injection of rAAV Into the Brain Newborn Mice

Serotype 1/2 rAAV-*SYN*-Venus virus stocks for neuronal labeling by the rAAV human synapsin promoter (Kügler et al., 2003) driven Venus protein (Pilpel et al., 2009) were generated using the rAAV serotype 1/2 packaging system (Klugmann et al., 2005; Tang et al., 2009), and purified by affinity chromatography purification (Smith et al., 2009). Virus infectious titers were determined in hippocampal primary cultures (1.0 × 10^7^–10^8^ infectious particle/ml). For neonatal injection (Pilpel et al., 2009), C57BL/6N pups were bilaterally virus-injected at P2 (four injection sites for each pup, bilateral: AP −1.0 mm, lateral ± 1.0 mm, depth 1.6 mm for lateral ventricle; AP–2.0 mm, lateral ± 1.0 mm, depth 1.5 mm for hippocampus; 1 μl purified rAAV*-SYN*-Venus/injection site).

### Animal Housing

After injection and recovery from anesthesia, pups were transferred to their home cages with their dams. Members of our research team monitored the physical condition of all animals 2–3 times per day until P8. At P28, offspring were separated from their mothers and housed with 1–2 sex-matched littermates/cage. At the age of 4 months, animals were transferred to individually ventilated cages in the animal room. The type II cages were installed in two-pipe system racks (Biozone). The cages were equipped with clean, fresh water and dust reduced wood bedding (Rettenmeier). The racks were individually ventilated by their own integrated ventilation systems (approx. negative pressure 5 Pa at 100 air exchanges/h). Room temperature in all animal rooms was kept at 20–24°C/relative humidity of 40–60% and constant lighting from 6:00 a.m. to 6:00 p.m. at 150–200 lux.

### Immunocytochemistry

Mice were anesthetized with isoflurane (Baxter Healthcare Corporation) and perfused intracardially with PBS (137 mM NaCl, 2.7 mM KCl, 4.3 mM Na_2_HPO_4_/ 2H_2_O, 1.4 mM KH_2_PO_4_; all from Sigma-Aldrich) and 4% paraformaldehyde (PFA, Merck) in PBS prior to decapitation. Brains were removed and after post-fixation in ice-cold 4% PFA for 2 h each brain was embedded in 2.5% agarose (Invitrogen)/PBS. Agarose embedded brains were sliced (70 μm sections) on a vibratome (VT1000s, Leica). To increase the contrast of the Venus signal in the thick vibratome sections, the Venus fluorescence was visualized together with its immunoreactivity by using a rabbit anti-GFP antibody (1:5,000, Millipore) and FITC-coupled anti-rabbit secondary antibody (1:200, Jackson ImmunoResearch). For the NeuN staining, mouse anti-NeuN antibody were used (1:1,000, Merck Millipore) and followed with Cy3-coupled anti-mouse secondary antibody (1:200, Jackson ImmunoResearch). Immunstaining was performed as described (Krestel et al., 2001).

### Maternal Care Monitoring

Six C57BL/6N and 3 FVB/N female mice were selected for this experiment (Table 1). The born pups were visually inspected and counted at the light cycle for 12–60 h before MMF-AFN treatment. At the age of about 60–72 h (P2) the pups of 2 C57BL/6N and 3 FVB/N litters received MMF-AFN anesthesia followed immediately by rAAV-*SYN*-Venus brain injections. Two naïve C57BL/6N litters served as controls. After the anesthesia and rAAV injection, the pups were returned back to their home cages with their dams and video-monitored from the top of the cage for 24 h. Two C57BL/6N litters were not included in the analysis due to the low number of surviving pups.

**Table 1:** Monitoring of C57BL/6N and FVB/N mice after birth.

Animal Strain/Litter	MMF-AFN+/rAAV injection+	New born pups	Remaining pups (at 24 h)	Remaining pups (at 48 h)	Remaining pups (at 72 h)	Remaining pups (at 96 h)
C57BL/6N 1	−/−	8	7	7	7	7
C57BL/6N 2	−/−	6	5	3	1	0
C57BL/6N 3	+/−	9	8	8	8	8
C57BL/6N 4	+/−	4	3	2	1	0
C57BL/6N 5	+/+	8	8	8	8	8
C57BL/6N 6	+/+	9	9	9	9	9
FVB/N 1	+/+	9	9	9	9	9
FVB/N 2	+/+	9	9	9	9	9
FVB/N 3	+/+	7	7	7	7	7

*Six C57BL/6N and three FVB/N female mothers were involved, and each litter is indicated with numbers of newborn pups. Observation of surviving newborn pups from each litter was done every 24 h for 4 days. Video-monitoring was done after MMF-AFN anesthesia combined with rAAV injection (at 60 h) for 2 h*.

### Behavioral Experiments

Behavioral experiments were performed with 3-month-old animals during the light cycle. Each behavioral test and pre-handling day, the home-caged mice were moved first to the animal test room for 1 h of habituation before the behavioral challenge started. Pre-handling with each mouse was performed for 3 days by the experimenter as described (Deacon, 2006b). The order of the following behavioral tests was performed according to earlier recommendations ranking from low to more stressful tests (van Gaalen and Steckler, 2000; McIlwain et al., 2001). For the behavioral test, mice were categorized into three groups: (i) MMF-AFN anesthesia + rAAV-*SYN*-Venus injection (MMF-AFN+V; *n* = 9); (ii) MMF-AFN anesthesia (MMF-AFN–V *n* = 10); and (iii) naïve controls (*n* = 7). In total four litters of C75BL6/N mice were selected for the behavioral tests. Before the behavioral test started, 2 MMF-AFN treated animals died, and 1 MMF-AFN + rAAV-*SYN*-Venus treated animal was excluded from the pre-handling due to hyperactivity (final experimental animal number: control animals without treatment and virus injection *n* = 7, MMF-AFN treated *n* = 8, MMF-AFN treated and virus-injected *n* = 8). The order of those 23 single-housed animals was randomized for the experimenter. The experimenter was blind to the history of each of those 23 C57BL6/N mice.

### Nesting Score

For the quantification of nesting behavior, mice were single-housed overnight in cages with 20 × 20 cm square sized white tissues as nesting material. At the next morning, nest construction was scored according to the following scheme: 1 = no nesting; 2 = some craters in bedding; 3 = nestlets mostly shredded but not identifiable nest site; 4 = an identifiable but flat nest; 5 = nestlets pulled to pieces and closed roof formed, covering the mouse completely. Higher scores in this task is associating with intact hippocampal processing (Deacon, 2006a).

### Tail Suspension Test

Mice were suspended by their tails for 6 min. The body and limb posture, as well as immobility and amount of movements, were assessed in 30 s intervals (Guyenet et al., 2010).

### Accelerating Rota-Rod

The Rota-Rod (# 47650; Ugo Basile) had five 3 cm diameter drums, suitably machined to provide grip. The mouse was held by the tail and released as soon as the mouse could grip the rotating rod, which was rotating at a speed of 4 rpm. The rod rotation was gradually accelerated until it reached the maximum of 40 rpm. If the mouse fell off the rod, the time was recorded as latency to fall of the Rota-Rod (Sprengel et al., 1998). The learning of motor coordination was recorded in two trial/day and in a 5 min/trial.

### Horizontal Bar Test

Animals were place on one end of an elevated Horizontal Bar (diameters were ranging in steps of from 32–6 mm). The time, it took each animal to reach the other end of the 50 cm long bar was recorded (Deacon, 2013).

### Elevated Plus-Maze

The self-constructed Elevated Plus-Maze was elevated 100 cm from the floor. The Plus-Maze was composed of two opposing 50 × 7 cm wide closed arms (7 cm high walls on each side) and two opposing 50 cm long open arms (Komada et al., 2008). The time in the open and closed arms was determined for each animal in one 5 min trial. Animals were placed in the center to start the trial.

### Dark-Light-Box

The Dark-Light-Box consists of an open, light illuminated white compartment (30 × 20 × 20 cm) and a neighboring 15 × 20 × 20 cm closed dark compartment. Both compartments were connected by an open door 3 × 3 cm. The mouse was placed in the middle of the light compartment facing away from the opening to the dark compartment. The number of transitions between the light and dark compartment as well as the total time spent on the dark compartment of an animal was measured in one 5 min test run (Crawley and Goodwin, 1980).

### Novel Object Exploration in the Open Field

Locomotor activity was assessed in an open field (self-constructed: wooden arena; 50 × 50 × 50 cm). Mice were placed in a corner of an open field and allowed to explore the novel environment for 10 min. The movement and the position of the animal was video-taped from the top. After 10 min, a novel object (LEGO TOY) was placed in the center of the open field. The time, which the mice spent in the center, was monitored during first 10 min (without object) and the next 5 min when the novel object was presented.

### T-Maze

The T-Maze test is a spatial working memory (SWM) test, analyzing the animals’ ability to alternate between a new unknown and a familiar environment based on their memory of the previously visited arms (Deacon and Rawlins, 2006). The T-shaped maze (self-constructed, of water-repellent panted wood) was composed of 40 cm long start arm, and two 20 cm long choice arms in a T-shape. All arms had a width of 10 cm and were surrounded by 10 cm high walls. Three days before the test and during the test, mice were food-deprived to keep them at about 85–90% of their original bodyweight. After three days of food depravation, the animals were pre-habituated to the T-Maze in two 10 min sessions with free access to small food rewards at the end of the goal arms. In the following days, the T-maze test was performed by six trials/day for four consecutive days, and an inter-trial interval of more than 1 h. Each trial was consisting of a sample and a choice run. In the sample run, the mouse was placed on the start arm of the T-maze and was directed to enter a rewarded goal arm (food pellet; TSE Systems) by blocking the other goal arm. After the reward was consumed by the mouse, the mouse was removed from the maze. Ten seconds later, the mouse was placed again in the start arm to perform the choice run. In this choice run both goal arms were accessible. Wild-types typically choose the unexplored arm of the T-Maze to search for the food reward in the choice run (Deacon and Rawlins, 2006). The identity of the sample arm for each trial was determined by random sequences. The percentage of the correct choice run was then calculated for the statistical analysis.

### Y-Maze

The Y-Maze test is for spatial reference memory (SRM), in which the animals learn to choose the rewarded goal arm based on the position of the extra maze cues (Bannerman et al., 2012). The Y-Maze was composed of three identical wooden, water-repellent painted arms (40 × 10 × 1.5 cm). Habituation and food deprivation were performed as described for the T-maze. The three arms of the Y-Maze were numbered 1–3, and different clearly visible cues surrounded the Maze. For each mouse, one arm was defined as target arm, the other two were used as pseudorandomized start arms in the following 64 trials composed of eight trials/day with a 12 min inter-trial interval. In each trial the mouse started in one of the two start arms, and the animal was rewarded with a small food pellet (TSE Systems) in the target arm. A full-body entries into one arm was counted as entry. After each trial, the arms of the Y-maze were cleaned with 80% ethanol. Before the next run was performed, the Y-maze was rotated by 120 degrees without changing the relative position of the target arm relative to the extra-spatial spatial cues in the experimental setup (Reisel et al., 2002).

### Statistics

Statistical analysis was performed using the statistics program Prism (Ver.7 for MacOSX). One-way ANOVA was performed for the body weight, Elevated Plus-Maze test, the Dark-Light-Box test and the open field activity test; two-way repeated measurement ANOVA was performed for Rota-Rad test, Horizontal Bar test, T-Maze and Y-Maze tests. *Post hoc* analysis was performed with Bonferroni *post hoc* tests.

## Results

### MMF-AFN Treatment of Neonatal Mice

First, we applied the MMF-AFN dosage protocol (Henke et al., 2004) to newborn mice at postnatal day 0 (P0). All pups (*n* = 14) were successfully anesthetized up to 30 min and recovered 3–5 min after antagonist injection. After the recovery from anesthesia, pups were placed back to their home cages. However, 12–24 h after MMF-ANF administration, all MMF-AFN treated neonatal mice had died. Similarly, the pups treated with MMF-AFN during the 24–48 h time window (P1) after birth (*n* = 15) did not survive the MMF-AFN treatment. Only when the MMF-AFN anesthesia was administrated during 48–72 h postnatal window (P2) all anesthetized pups (*n* = 10) recovered from the temporal MMF-AFN anesthesia. It is known that the MMF-AFN administration might affect the animals’ cardiorespiratory system (Henke et al., 2005). Our observation with a small pilot group of P0 and P1 mouse shows that the MMF-AFN anesthesia is lethal for the neonatal animals when applied in 0–48 h after birth (P0–P1), possibly due to cardiorespiratory failures caused by the drug during this sensitive time window, which is necessary for the establishment of autonomously operating vegetative functions.

### Neonatal rAAV Delivery Based on MMF-AFN Anesthesia

Our study was aimed to explore whether MMF-AFN-anesthetized neonatal mice could be subjected to rAAV delivery by stereotactic brain injections.

**Figure 1:**
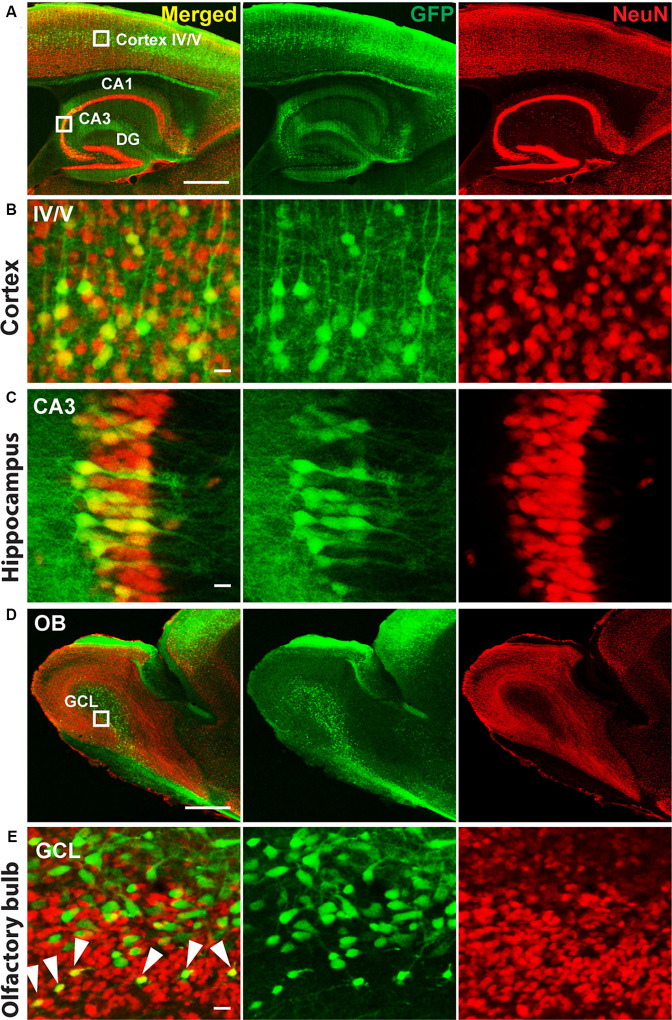
Immunohistochemistry of P2 recombinant adeno-associated virus (rAAV)-*SYN*-Venus transduced mouse brain in adulthood. Confocal images of a sagittal brain section 6 month after the P2 rAAV injection under Medetomidine, Midazolam, and Fentanyl (MMF)-Atipamezole/Flumazenil/Naloxone (AFN) anesthesia. Co-staining of Venus (green, visualized with FITC-coupled GFP-antibody, to intensify the signal for Venus expression) and NeuN (red, visualized with Cy3-coupled antibody are depicted. **(A)** Transduced mouse cortex and hippocampal regions. **(B,C)** High magnification images of the boxed cortex layer IV/V region and boxed hippocampal CA3 region shown in **(A)**. **(D)** Overview images of the olfactory bulb from a rAAV-*SYN*-Venus infected mouse. **(E)** Confocal images of the infected olfactory bulb. Green only cells are showing bigger cell size and do not co-localize with NeuN signals, indicating the immature stage of the infected cells. NeuN positive green cells are localized in the mature granule cell layer (GCL) indicated by white arrows, demonstrating the migration and maturation of the infected cells. Co-staining of Venus (green) was visualized with FITC-coupled GFP antibody and NeuN (red) with Cy3-coupled anti-body. IV/V, cortical layer IV/V; DG, dentate gyrus; CA1–3, hippocampal layers 1–3; OB, olfactory bulb; GLC, granule cell layer; scale bars in **(A,D)** = 500 μm; in **(B,C,E)** = 10 μm.

Therefore, we delivered, for neuronal specific expression, infectious rAAV-*SYN*-Venus virus particles during the MMF-AFN treatment by bilateral rAAV injections into hippocampi and lateral ventricles of C57BL/6N neonates at P2. Six months post virus injection, we found global Venus immunosignals predominantly in hippocampal and cortical neurons and in cells of the olfactory bulb (Figure 1). Strongest Venus expression was found in in somata of pyramidal CA3 cells (Figure 1C) but also in cortical layer IV/V neurons (Figures 1A,B), possible due to virus spread into cortical areas during the hippocampal and lateral ventricle injections of rAAV-*SYN*-Venus. Immuno-reactive Venus expressing cells were also found in the mitral and granule cell layer (GCL) of the main olfactory bulb (Figure 1D). Most Venus expressing cells in the center of the GCL do not overlap with NeuN signals. Those cells display bigger cell size compared to NeuN positive cells, suggesting that they might represent progenitors or migrating neural stem cells derived from rAAV-*SYN*-Venus-transduced subventricular zone (SVZ) stem cells and that the human synapsin promoter fragment of the rAAV-*SYN*-Venus is already active in those neuronal progenitor cells. NeuN and anti-GFP positive neurons are located outside the center of the olfactory bulb. Most likely, they represent terminally-differentiated mature granule cells derived from rAAV-transduced migrating neuronal stem cells of the SVZ (Figure 1E).

### Monitoring and Comparison of Maternal Care From C57BL/6N and FVB/N Dams After MMF-AFN Administration and rAAV Delivery

Maternal behavior is the pattern of care given by mothers to their offspring. Therefore, we used video-monitoring to compare the maternal care of FVB/N and C57BL/6N dams. Before the MMF-AFN administration and rAAV injection, two litters of C57BL/6N animals were excluded from this analysis due to the low number of surviving pups, 60 h post-delivery (Table 1: litter 2 and litter 4). During the 36 h recording, before and 24 h after MMF-AFN, MMF-AFN+Virus and No-treatment, the maternal care behaviors of all dams, such as nest building, gathering pups together into the nest and keeping pups warm were comparable for all dams. All dams were able to build their nests and retrieve/remain their pups in the nest (data not shown). Importantly, none of the pups died after the MMF-AFN and the MMF-AFN+Virus injection at P2 (Table 1).

### Behavior Analysis of MMF-AFN Anesthetized Animals

**Figure 2:**
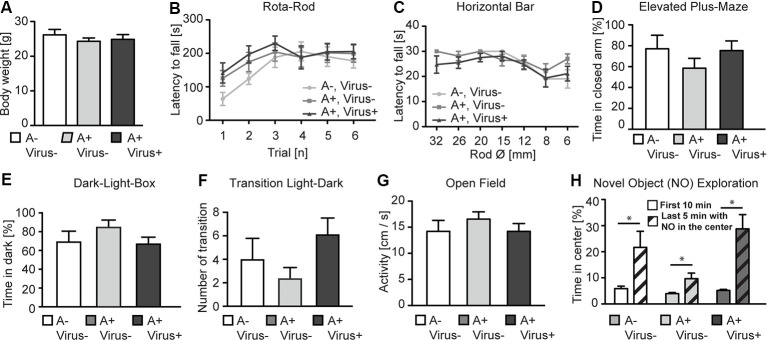
Behavioral phenotyping of adult P2 MMF-AFN-anesthetized mice. **(A)** The body weight of adult P2 MMF-AFN-anesthetized animals, including rAAV-injected animals, were comparable to the body weight of controls. No significant differences were observed among all three groups. **(B)** In Rota-Rod adult, P2-MMF-AFN anesthetized mice, including rAAV-injected mice, showed the same performance as their naïve controls. Animals of all three groups were able to improve their motor coordination over trials. **(C)** Similarly, in the Horizontal Bar test, no difference in the performance was observed among animals of the three groups. **(D)** In the Elevated Plus-Maze test, all animal groups exhibited comparable level of anxiety-related behavior as measured by the relative time that the animals spent in the closed arms of the maze. **(E)** In the Dark-Light-Box test, all animal groups showed comparable time spent in the dark compartment, and **(F)** similar number of transitions from the light to the dark compartment. **(G)** In the open field test, no difference in locomotion activity could be observed in 10 min recordings of the animals from all three groups during the first 10 min in the novel open field. **(H)** The increased novel object exploration was not altered in P2-MMF-AFN anesthetized as well as P2-MMF-AFN-rAAV-infected adult animals. All mice spent more time in the center of the open field after the novel object was introduced during the last 5 min into the center of the open field. A–Virus– (naïve animals); A+Virus– (MMF-AFN treatment at P2); A+Virus+ (MMF-AFN coupled with rAAV injection in the brain at P2). *=Paired *t*-test: pA−,V− = 0.0395; pA+,V− = 0.0371; pA+,V+ = 0.0037.

To analyze whether the MMF-AFN anesthesia in P2 pups will lead to behavioral impairments in the adults, all P2 test animals were separated from their mothers and caged in sex-matched groups at P28. Offspring from C57BL/6N dams were randomly categorized into three groups: untreated, naïve control animals (*n* = 7), MMF-AFN–Virus (*n* = 8) and MMF-AFN+Virus (*n* = 8) animals. Eleven weeks post-delivery, mice of those three groups were single-housed for 1 week before the pre-handling and the behavioral tests started. When analyzed for the home-cage behavior, mice of all three groups exhibited regular nesting score and tail suspension (data not shown), and had comparable body weight at the age of 12 weeks (Figure 2A; one-way ANOVA, *F*_(2,20)_ = 0.5006, *p* = 0.6136). Motor coordination of the mice was analyzed in the accelerating Rota-Rod and the Horizontal Bar tests. All animals of the three cohorts performed equally well in the Rota-Rod, and Two-way repeated measurement ANOVA showed no significant difference among all groups (Figure 2B; Two-way repeated measurement ANOVA, *F*_(2,100)_ = 0.6623, *p* = 0.5266). Similar results were found in the Horizontal Bar test (Figure 2C; Two-way repeated measurement ANOVA, *F*_(2,120)_ = 1.228, *p* = 0.3141). Thus, neither the home-cage behavior nor the simple or complex motor coordination tests provided any indication for potential long-term general neuronal dysfunction induced by MMF-AFN treatment and/or the combined rAAV injection at P2.

The anxiety-related behavior was monitored in the Elevated Plus-Maze and the Dark-Light-Box. The animals from MMF-AFN anesthetized with rAAV-injected group and only MMF-AFN anesthetized group showed no increased anxiety-related behavior when compared to untreated mice in Elevated Plus-Maze. The time the mice spent in the closed arms was not significantly different among mice of the three groups (Figure 2D; one-way ANOVA, *F*_(2,20)_ = 0.9771, *p* = 0.3937). Similarly, in the Dark-Light-Box, the total time spent in the dark compartment was comparable (Figure 2E; one-way ANOVA, *F*_(2,20)_ = 1.384, *p* = 0.2736) and the number of transitions between the light and dark compartment showed no significant difference among groups (Figure 2F; one-way ANOVA, *F*_(2,20)_ = 1.945, *p* = 0.1691). To further investigate the response to novel environments and objects, locomotion and object exploration were monitored in the open field (Figure 2G). The open field activities before introducing a novel object were similar in all three groups. One-way ANOVA and the *post hoc* test (Bonferroni’s Multiple Comparison Test) showed no significant difference among three groups (Figure 2G; one-way ANOVA, *F*_(2,20)_ = 0.5163, *p* = 0.3064), demonstrating normal locomotion of the P2 MMF-AFN-treated animals in comparison to naïve mice. After introducing a novel object (NO) in the center of the open field, all mice spent more time in the center to explore this object compared the time that they spent in the outer quadrants of the open field (Figure 2H; Paired *t*-test, p_A−,V−_ = 0.0395; p_A+,V−_ = 0.0371; p_A+,V+_ = 0.0037). Thus, the NO exploration ability was also not affected by the MMF-AFN treatment, combined with injections at P2.

### Spatial Working (SWM) and Spatial Reference Memory (SRM) Were Not Affected by the MMF-AFN Treatment and rAAV Delivery at P2

**Figure 3:**
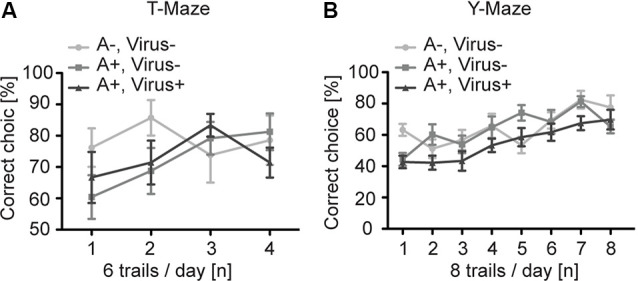
Spatial learning in the T- and Y-Maze of adult P2 MMF-AFN anesthetized mice. **(A)** In T-Maze task, the performance of adult mice of the three test groups was comparable. A−Virus– (naïve animals); A+Virus– (MMF-AFN treatment at P2); A+Virus+ (MMF-AFN treatment coupled with rAAV injection in the brain at P2). **(B)** Similarly, in the Y-Maze all animals shown in panel **(A)** were able to learn the spatial position or the rewarded arm. There was no significant difference in the daily performance, and no difference was detected among all groups.

To analyze higher cognitive function in P2-MMF-AFN anesthetized and rAAV-injected mice, we first examined the SWM in the T-Maze in the 3 months old P2-MMF-AFN anesthetized and rAAV-injected mice (Reisel et al., 2002). Independent of the pharmacological and neurosurgical intervention at P2, all tested C57BL/6N mice were able to perform this task above chance level (50%). The two-way repeated measurement ANOVA showed no significant differences among all groups (Figure 3A; two-way repeated measurement ANOVA, interaction *F*_(6,57)_ = 1.074, *p* = 0.3886, treatment *F*_(2,19)_ = 1.229, *p* = 0.3148, and trials *F*_(3,57)_ = 1.525, *p* = 0.2179). *Post hoc* tests (Bonferroni test) demonstrated also no difference on each experimental day between either two of the three groups (data not shown). Thus, SWM was not changed by rAAV injections under MMF-AFN anesthesia at P2.

For the analysis of the SRM we employed the Y-Maze task (Bannerman et al., 2012). Despite of the different treatments of mice at P2, all animals of the three groups demonstrated an improved learning curve with a significant learning during eight successive experimental days (Figure 3B; two-way repeated measurement ANOVA, day trials *F*_(7,133)_ = 15.03, *p* < 0.0001). And two-way repeated measurement ANOVA showed no difference among all groups (*F*_(2,19)_ = 2.049, *p* = 0.1563). *Post hoc* tests (Boferroni test) indicated no difference on each experimental day between either two of the three groups (data not shown). Taken together, both MMF-AFN administration and virus treatment of the animals at the neonatal stage did not affect behaviors in their adulthood.

## Discussion

In this study putative behavioral side effects of MMF-AFN anesthesia, combined with concurrent rAAV injection into the developing brain of neonates were assessed in their adulthood. For this purpose, pups of four C57BL/6N litters were anesthetized at P2, and half of the anesthetized pups received bilateral rAAV brain injections. At the age of 3 months, the matured mice were subjected to standardized behavioral tests for motor coordination, anxiety-related behavior, novel object recognition, SWM and SRM formation. In our study the neonatal MMF-AFN and brief neuosurgical intervention did not result in sever basic and cognitive behavioral impairments in animals when they reach their adulthood.

Females from the FVB/N strain were employed to compare the maternal care behaviors with that of C57BL/6N dams. Our video-monitoring showed that the C57BL/6N dams exhibit less maternal care towards their pups, since two out of six C57BL/6N dams were not able to gather and maintain their offspring within the first 60 h after birth (Table 1). However, in those litters of C57BL/6N dams that showed maternal care within the first 60 h after birth, almost all pups survived the anesthesia given at P2, indicating that the MMF-AFN combined with rAAV injection has no major impact on the early mortality of pups in both C57BL/6N and FVB/N mouse strains. Two out of 19 MMF-AFN anesthetized animals (10.5%) were not able to survive until adulthood. From our result, a mortality rate of 10% within the first 4 weeks after birth is similar to the mortality of mice raised and housed in animal facilities.

Even though the MMF anesthesia followed with AFN-induced recovery could not be applied to P0 and P1 animals, which is most likely due to due to cardioresperitory side effects (Henke et al., 2005), the P2 MMF-AFN and rAAV-infected mice, showed a global rAAV gene transfer in the adult mouse brains, including large areas of the cortex, hippocampus and olfactory bulb, which is comparable to the rAAV injection in the hippocampus and SVZ of cryo-anesthetized mice at P0 (Berkel et al., 2012). The strongest expression of the viral-transduced Venus was again found in CA3 pyramidal neurons of the stratum pyramidale of the hippocampus and in the cortical layer IV/V. In the olfactory bulb, specifically the granular cells, showed Venus expression. Since the olfactory bulb was not directly targeted by rAAV injections, it is reasonable to assume that Venus-expressing granular cells in the olfactory bulb are derived from rAAV-infected neuronal stem cells from lateral ventricles (Pilpel et al., 2009). Currently, it cannot be resolved whether the transduced Venus expression in cortical neurons is due to spill-over-released rAAV during the injection through the cortex, or relies on infected precursor cells, since similar rAAV-mediated gene expression pattern in the frontal cortex, hippocampus and ventricles was found when the P0 injections were directed to the all three brain regions (Pilpel et al., 2009). Further studies are necessary to solve this question.

Unlike the previous study showing that cryoanesthesia of neonatal mice can cause slight increase of anxiety level in adulthood (Richter et al., 2014), our results showed that the brief MMF-AFN administration combined with crude neurosurgery cannot be recognized in behavioral deficits in adult mice. Our standard behavioral tests indicated that the neonatal MMF-AFN anesthesia permits a direct behavioral assessment of P2 virus-transduced genes later in adult animals. Furthermore, it demonstrates that the MMF-AFN administration could be used as an alternative approach to hypothermia for brain specific, neonatal rAAV gene transduction in experimental research.

## Data Availability Statement

The raw data supporting the conclusions of this article will be made available by the authors, without undue reservation.

## Ethics Statement

The animal study was reviewed and approved by the Animal Ethics Committee of the Max Planck Institute for Medical Research Heidelberg.

## Author Contributions

WT conducted the experiments and analyzed the data and contributed to writing of the manuscript. UZ designed and provided the anesthesia protocol. RS contributed to the design of the experiments and writing of the manuscript.

## Conflict of Interest

The authors declare that the research was conducted in the absence of any commercial or financial relationships that could be construed as a potential conflict of interest.
